# An Origin-of-Life Reactor to Simulate Alkaline Hydrothermal Vents

**DOI:** 10.1007/s00239-014-9658-4

**Published:** 2014-11-27

**Authors:** Barry Herschy, Alexandra Whicher, Eloi Camprubi, Cameron Watson, Lewis Dartnell, John Ward, Julian R. G. Evans, Nick Lane

**Affiliations:** 1 Department of Genetics, Evolution and Environment, University College London, London, UK; 2 Space Research Centre, Department of Physics and Astronomy, University of Leicester, Leicester, UK; 3 Department of Biochemical Engineering, University College London, London, UK; 4 Department of Chemistry, University College London, London, UK

**Keywords:** Serpentinization, Alkaline vent, Hydrothermal, CO_2_ reduction, Thermophoresis

## Abstract

Chemiosmotic coupling is universal: practically all cells harness electrochemical proton gradients across membranes to drive ATP synthesis, powering biochemistry. Autotrophic cells, including phototrophs and chemolithotrophs, also use proton gradients to power carbon fixation directly. The universality of chemiosmotic coupling suggests that it arose very early in evolution, but its origins are obscure. Alkaline hydrothermal systems sustain natural proton gradients across the thin inorganic barriers of interconnected micropores within deep-sea vents. In Hadean oceans, these inorganic barriers should have contained catalytic Fe(Ni)S minerals similar in structure to cofactors in modern metabolic enzymes, suggesting a possible abiotic origin of chemiosmotic coupling. The continuous supply of H_2_ and CO_2_ from vent fluids and early oceans, respectively, offers further parallels with the biochemistry of ancient autotrophic cells, notably the acetyl CoA pathway in archaea and bacteria. However, the precise mechanisms by which natural proton gradients, H_2_, CO_2_ and metal sulphides could have driven organic synthesis are uncertain, and theoretical ideas lack empirical support. We have built a simple electrochemical reactor to simulate conditions in alkaline hydrothermal vents, allowing investigation of the possibility that abiotic vent chemistry could prefigure the origins of biochemistry. We discuss the construction and testing of the reactor, describing the precipitation of thin-walled, inorganic structures containing nickel-doped mackinawite, a catalytic Fe(Ni)S mineral, under prebiotic ocean conditions. These simulated vent structures appear to generate low yields of simple organics. Synthetic microporous matrices can concentrate organics by thermophoresis over several orders of magnitude under continuous open-flow vent conditions.

## The Origins of Biochemistry

Does the biochemistry of modern cells offer any insight into its own origins? There are good reasons to seek congruence between the origins of biochemistry and particular early Earth environments. de Duve (2005) has asked how biological catalysts, whether enzymes or ribozymes, might have first arisen, to which his “only scientifically plausible explanation” was “by selection”. If so, the first biological catalysts must have been selected in the context of protometabolism, meaning that they enhanced a process that occurred spontaneously, driven by natural disequilibria and catalysed by inorganic catalysts. Selection therefore imposes a link between protometabolism and metabolism, providing good grounds for seeking just such a link. This argument is at least parsimonious, and advocates that we should look to life itself for clues to how life arose (Martin et al. 2014). One factor in particular distinguishes living cells from conventional synthetic chemistry as practised by humans: biochemistry is fundamentally vectorial. It has structure and direction in space, as pointed out by Peter Mitchell from the late 1950s onwards (Mitchell 1959, 1961, 1966). Most importantly, in all known autotrophic bacteria and archaea, carbon and energy metabolism is driven by electrochemical ion (generally proton) gradients across membranes, Mitchell’s chemiosmotic coupling (Maden 1995; Stetter 2006; Lane et al. 2010). Geochemical systems with analogous vectorial chemistry could therefore offer valuable insights into the origin of life.

Searching for congruence between geochemistry and biochemistry may be reasonable, but which aspects of biochemistry are genuinely ancient, and which are more recent adaptations to specific (potentially ancient) environments? It is nearly impossible to build a phylogenetic tree of life to reconstruct the biochemistry of the Last Universal Common Ancestor (LUCA), as frequent lateral gene transfer (LGT) in bacteria and archaea has confounded the order of deep branches (Doolittle 1999; Doolittle and Bapteste 2007; Dagan and Martin 2007; Sousa et al. 2013; Nelson-Sathi et al. 2014). However, deep biochemical differences between the domains of life do give some insights. Because eukaryotes are secondarily derived from an endosymbiosis between an archaeal host cell and bacterial endosymbionts (Cox et al. 2008; Williams et al. 2013), LUCA was the common ancestor of bacteria and archaea (Dagan et al. 2010). While bacteria and archaea share a number of fundamental traits including transcription, ribosomal translation, aspects of amino acid biosynthesis and chemiosmotic coupling using a membrane-integral ATP synthase, several other fundamental traits are shockingly different (Martin and Russell 2003; Sousa et al. 2013). For example, most enzymes involved in DNA replication are not homologous in bacteria and archaea (Edgell and Doolittle 1997; Leipe et al. 1999).

Most importantly, the cell membrane and cell wall are radically different in their chemistry and stereochemistry (Koga et al. 1998; Peretó et al. 2004), as are the enzymes for lipid biosynthesis (Martin and Russell 2003; Peretó et al. 2004; Lombard et al. 2012). Biochemical pathways such as glycolysis (Say and Fuchs 2010), and heme and quinone synthesis (Sousa et al. 2013) are also distinct, albeit with some confounding LGT. Such considerations imply that fermentation and respiration, or more specifically the heme and quinone-dependent proteins used for respiratory ion pumping, arose independently in the bacteria and archaea, but see Ducluzeau et al. (2014) for a contrary view.

These paradoxical properties could be resolved if LUCA depended on natural (geochemically sustained) proton gradients to drive carbon and energy metabolism, a lifestyle demanding membranes extremely leaky to protons (Lane and Martin 2012; Sojo et al. 2014). This requirement for leaky membranes could explain the divergence in other traits that might have coevolved later with membranes (see Sojo et al. 2014) notably DNA replication (in which the replicon is attached to the membrane in most bacteria), and cell wall synthesis, which requires membrane-integral export machinery.

Despite these stark disparities, some clues do exist to early biochemistry. Strikingly, there are only six known pathways of carbon fixation across all life (Fuchs 2011), but just one of these, the acetyl CoA pathway, is found in both archaea (methanogens) and bacteria (acetogens), albeit with some striking differences between them (Maden 2000; Martin 2012; Sousa and Martin 2014). Neglecting these differences, several other factors testify to the antiquity of the acetyl CoA pathway. It is the only exergonic pathway of carbon fixation, drawing on just H_2_ and CO_2_ as substrates to drive both carbon and energy metabolism (Fuchs and Stupperich 1985; Ragsdale and Pierce 2008; Ljungdahl 2009); what Everett Shock has called “a free lunch you’re paid to eat” (Shock et al. 1998). It is short and linear, with just a few steps leading from H_2_ and CO_2_ to acetyl CoA and pyruvate, the gateway to intermediary metabolism (Fuchs 2011; Morowitz et al. 2000), thereby avoiding the problem of sequentially declining yields with non-enzymic cycles that might have precluded an abiotic reductive TCA cycle (Orgel 2008). It is replete with Fe(Ni)S proteins, in which the inorganic cofactors that actually catalyse the key reactions in proteins such as ferredoxin and hydrogenases have structures essentially identical to FeS minerals such as greigite and mackinawite (Eck and Dayhoff 1966; Russell and Martin 2004; Ragsdale and Kumar 1996; Cody 2004; Major et al. 2004; Baymann et al. 2003; Kim et al. 2013; Harel et al. 2014). Finally, despite being exergonic overall, the acetyl CoA pathway is strictly dependent on chemiosmotic coupling in both methanogens and acetogens (Thauer et al. 2007; Poehlein et al. 2012). In the case of methanogens, the H^+^ (or Na^+^) gradient drives ferredoxin reduction via the Fe(Ni)S membrane protein, Ech (Kaster et al. 2011, Buckel and Thauer 2013).

All these factors—H_2_, CO_2_, Fe(Ni)S catalysis and electrochemical proton gradients—point to one very specific environment on the early Earth as the cradle of life: alkaline hydrothermal vents (Martin et al. 2014).

## Alkaline Hydrothermal Vents

### Early Vents as Electrochemical Reactors

Russell and colleagues (Russell et al. 1988, 1989, 1993, 1994; Russell and Hall 1997) predicted the existence and properties of deep-ocean alkaline hydrothermal systems more than a decade before their discovery, pointing out their suitability as natural electrochemical reactors capable of driving the origin of life. While fossil vent systems had been reported in Ireland (Boyce et al. 1983), the discovery of the first active submarine system, Lost City hydrothermal field (Kelley et al. 2001, 2005), was remarkable in that its properties corresponded almost exactly to those postulated by Russell et al. (1993). Lost City is powered by a process called serpentinization, the exothermic reaction of ultramafic minerals from the upper mantle, in particular olivine, with water (Bach et al. 2006; Sleep et al. 2004; Martin et al. 2008; Russell et al. 2010). This reaction produces large volumes of H_2_ (the presence of the mineral awaruite in some serpentinizing systems indicating as much as 200 mM (McCollom and Bach 2009) dissolved in warm (45–90 °C) alkaline (pH 9–11) fluids containing magnesium hydroxides (Kelley et al. 2001, 2005). Alkaline vents do not form chimneys, as in black smokers (and indeed do not normally ‘smoke’ at all) but rather are labyrinthine networks of interconnected micropores bounded by thin inorganic walls, through which hydrothermal fluids (and ocean waters) percolate.

Such vents should have been more common on the early Earth, as the mantle was less differentiated from the crust, hence ultramafic minerals could have been found across much of the ocean floor (Fyfe 1994; Jaffrés et al. 2007; Shields and Kasting 2007). In contrast, ultramafic minerals are mostly exposed close to the mid-ocean spreading centres today (Schrenk et al. 2013). Alkaline vents are highly stable geological systems; Lost City is estimated to be about 100,000-years old (Ludwig et al. 2005), which as noted by Russell, is 10^17^ microseconds, a time unit more consonant with chemistry. That gives plenty of time for abiotic chemistry to develop, especially if early vents were indeed contiguous across the ocean floor (Sleep 2010; Shields and Kasting 2007). Moreover, the fact that olivine and water are both abundant in space (de Leeuw et al. 2010), and so presumably on all wet, rocky Earth-like planets, implies that equivalent conditions could be projected to occur on as many as 40 billion exoplanets in the Milky Way alone (Lane 2015).

Lost City is composed of carbonate minerals, mostly aragonite, and magnesium hydroxide, brucite, (Kelley et al. 2001, 2005) but this is unlikely to represent the composition of ancient vents. That difference is critical and relates not to serpentinization as a process (which should have been the same), but to ocean chemistry in the Hadean and Archaean, around 4 billion years ago (Pinti 2005). There were two critical differences: oxygen was absent (Bekker et al. 2004; Kasting 2013); and the CO_2_ concentration in the oceans was substantially higher (although there is little consensus on how much higher; see Russell and Arndt 2005; Sleep 2010; Arndt and Nisbet 2012). Anoxia is necessary for both thermodynamic and kinetic reasons. Thermodynamic, because the reaction between H_2_ and CO_2_ is only favoured under anoxic conditions (Amend et al. 2013); and kinetic, because the solubility of catalytic transition metals, notably Fe^2+^ and Ni^2+^ is much greater when oceans are anoxic (Russell and Arndt 2005; Arndt and Nisbet 2012). That the Hadean oceans were indeed replete in Fe^2+^ and Ni^2+^ (derived from volcanic systems such as black smokers) are indicated by the precipitation of vast banded-iron formations throughout the Archaean (Anbar and Holland 1992; Zahnle et al. 2007). The great availability of transition metals (along with bisulphide ions within alkaline vents; Nitschke and Russell 2009) must have resulted in the precipitation of catalytic Fe(Ni)S minerals such as mackinawite and greigite in the walls of the vents themselves; but equivalent catalytic Fe(Ni)S minerals are not found in modern vents. In early vents then, H_2_-rich hydrothermal fluids must have percolated through labyrinths of micropores bounded by thin inorganic walls containing catalytic Fe(Ni)S minerals (Nitschke and Russell 2009; Lane and Martin 2012).

The higher CO_2_ concentration in Hadean oceans should have increased carbon availability (modern alkaline hydrothermal vents are often carbon limited, from carbonate precipitation and removal by living cells; Proskurowski et al. 2008; Bradley et al. 2009) and lowered the pH of the oceans, probably to around pH 5–7 (Arndt and Nisbet 2012). That could have produced pH gradients of 5 or 6 pH units between the alkaline hydrothermal fluids and acidic oceans. While mixing could prevent such steep gradients being juxtaposed across single barriers, laminar flow in elongated hydrothermal pores does make it feasible for sharp gradients of several pH units to exist across distances of a few micrometres.

### The Driving Force for Organic Synthesis

Steep natural proton gradients across thin catalytic Fe(Ni)S barriers could theoretically promote organic synthesis by lowering the energetic barrier to CO_2_ reduction (Lane 2014; Yamaguchi et al. 2014). Amend and McCollom (2009) calculated that anoxic alkaline hydrothermal conditions (between 25 and 125 °C) are thermodynamically conducive to the synthesis of total cell biomass (i.e. amino acids, fatty acids, carbohydrates, nucleotides) from H_2_ and CO_2_. Nonetheless, experimental attempts to drive the reaction of H_2_ and CO_2_ using Fe(Ni)S catalysts have proved unsuccessful, even at high pressures (Shock and Canovas 2010), as the reduction potential of the H_2_/2H^+^ couple is not sufficiently low to reduce CO_2_ to CO, formate (HCOO^−^), formaldehyde (HCHO) or similar organics with equivalent reduction potentials (Lane 2014; Lane and Martin 2012).

**Fig. 1:**
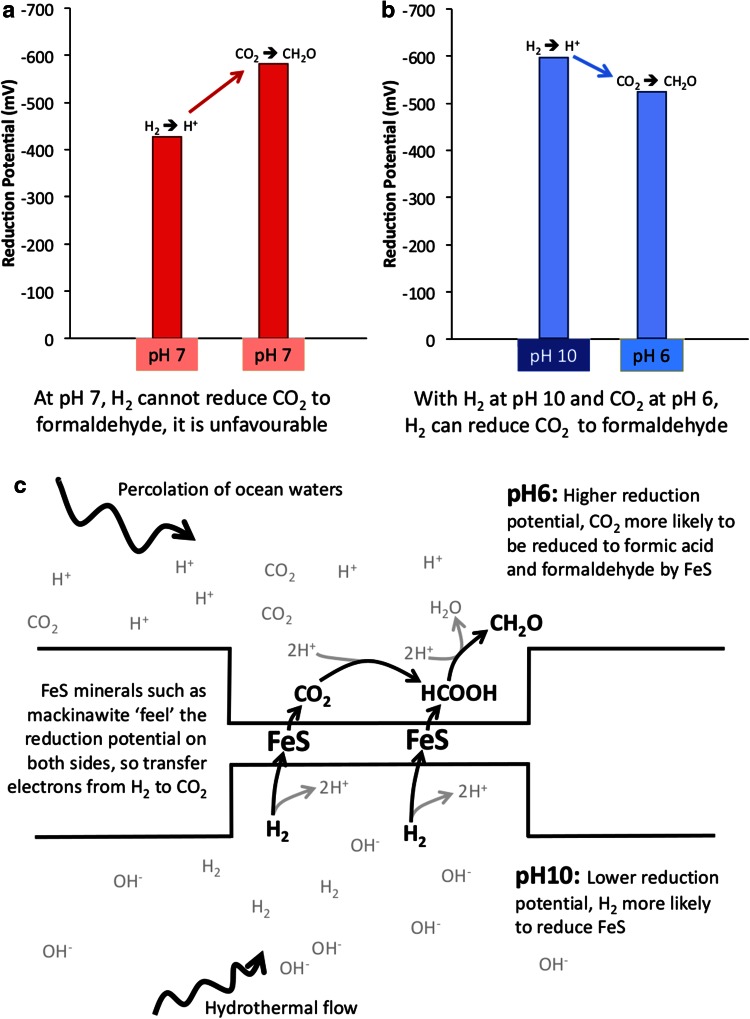
**a** Standard reduction potentials of H_2_ and CO_2_ at pH 7. Transfer of electrons from H_2_ to CO_2_ is unfavourable as the reduction potential for CO_2_ at this pH is lower (more negative) than H_2_. **b** With H_2_ dissolved in waters at pH 10 and dissolved CO_2_ in waters at pH 6 however, the reduction potential for CO_2_ becomes higher (more positive) than that of H_2_ making the reduction of CO_2_ favourable. This would theoretically allow for the reduction of CO_2_ to form organic compounds such as formate, formaldehyde, methanol and methane. **c** How acid and alkaline fluids could interact inside hydrothermal vents across thin semi-conducting Fe(Ni)S walls, leading to the reduction of CO_2_ to formaldehyde via formate

A clue might lie in the strict dependence of methanogens and acetogens on proton gradients to drive CO_2_ reduction (Buckel and Thauer 2013). In methanogens, the membrane-integral energy converting hydrogenase (Ech) uses the proton-motive force to reduce ferredoxin directly, which in turn reduces CO_2_, ultimately to a methyl group (Buckel and Thauer 2013). Ech could conceivably utilise proton gradients to modulate pH within the active site of the enzyme, thereby altering the reduction potential locally. Whenever protons are involved in a reduction, the reduction potential depends on pH, falling by ~59 mV per pH unit rise, according to the Nernst equation (Nicholls and Ferguson 2013). Such pH dependence is true of both H_2_ and CO_2_, hence at any particular pH, the reduction remains equally difficult (Fig. 1a). However, in alkaline vents, H_2_ is dissolved in hydrothermal fluids at pH 10, whereas CO_2_ is dissolved in ocean waters at pH 6. This sharp difference should modulate both reduction potentials sufficiently to drive the reduction of CO_2_ with H_2_. If fluids of pH 6 and 10 are juxtaposed across a thin semi-conducting Fe(Ni)S barrier, it should be possible in principle to reduce CO_2_ to CO, HCOO^−^ and even HCHO (Fig. 1b).

Once the energetic barrier to CO_2_ reduction has been overcome, the ensuing steps of the acetyl CoA pathway are exergonic, and in methanogens and acetogens drive carbon and energy metabolism via acetyl CoA and ATP, respectively (Fuchs 2011). An abiotic equivalent of this pathway could arguably generate the reactive thioester methyl thioacetate, a simple analogue of acetyl CoA, which has been synthesised from CO and CH_3_SH by Huber and Wäctershäuser (1997) using Fe(Ni)S catalysts. In modern cells, acetyl CoA can be phosphorylated without an enzyme to form acetyl phosphate, a reactive acyl phosphate that could act as an abiotic equivalent to ATP, with a higher phosphorylating potential (AcP ∆G′_0_ = −43 kJ mol^−1^, ATP ∆G′_0ADP_ = −31 kJ mol^−1^), providing a source of metabolic energy for phosphorylation and condensation to form polymers such as polypeptides and RNA (de Duve 1988, 1995; Martin and Russell 2007; Lane and Martin 2012). Overall, substrate-level phosphorylation produces acetyl phosphate, as argued by Ferry and House (2006), but in this case the whole process is driven by natural proton gradients.

Further phosphorylation and condensation reactions are only favoured if the concentration of monomers is high. That is possible, despite the anticipated low yields of most of these reactions, because alkaline hydrothermal vents should provide a dynamic concentration mechanism known as thermophoresis (Braun and Libchaber 2002; Baaske et al. 2007). Convection currents and thermal diffusion across the interconnected microporous matrix of alkaline vents produce thermal gradients that can concentrate organic molecules in the cooler regions. In closed experimental systems, even small thermal gradients (2.3–4.4 K) concentrate large molecules, notably DNA (Reineck et al. 2010) and RNA (Mast and Braun 2010; Mast et al. 2013), while fatty acids can be concentrated sufficiently to precipitate into vesicles (Budin et al. 2009). Thermophoresis is predicted to concentrate organics in open systems such as alkaline vents, but this has not previously been tested.

At a later stage, some form of compartmentalization is also crucial for selection to act on groups of replicators (e.g. RNAs) encoding functions such as metabolism and cooperation, rather than replication speed alone, which invariably leads to the formation of ‘Spiegelman’s monsters’ (Mills et al. 1967; Branciamore et al. 2009). The natural inorganic compartments in alkaline vents could facilitate not only the concentration of organics by thermophoresis, but also the beginnings of selection for metabolism (Branciamore et al. 2009; Koonin and Martin 2005). The two processes combined could potentially drive the replication of simple organic vesicles composed of mixed amphiphiles enclosing primitive replicators within vent pores (Budin et al. 2009; Mauer and Monndard 2011). Such vesicles are capable of growth and division, while retaining RNA (Hanczyc et al. 2003; Mansy et al. 2008) and are *en route* to the known end-point, modern cells with lipid membranes.

In sum, alkaline hydrothermal vents have the potential to drive the origins of biochemistry from H_2_ and CO_2_ using natural proton gradients and Fe(Ni)S minerals, in a manner remarkably analogous to the acetyl CoA pathway in methanogens and acetogens. Modern vent systems cannot replicate this chemistry, as modern oceans are aerobic, so extant vent systems lack Fe(Ni)S catalysts; they are also depleted in CO_2_, starving them of carbon and diminishing natural proton gradients; and any abiotic carbon chemistry is complicated by the presence of living cells. We have therefore built a simple bench-top reactor, which operates in an anaerobic hood, to simulate pertinent conditions in alkaline hydrothermal vents and test whether such conditions could drive the origins of biochemistry.

## An Electrochemical Reactor to Simulate Alkaline Hydrothermal Vents

We report the construction and preliminary testing of a continuous, open-flow, bench-top reactor to investigate the potential of alkaline hydrothermal systems to drive the origins of biochemistry. We explore

(i) The potential of alkaline hydrothermal vents to form simple organic molecules, most importantly formaldehyde (HCHO), by reducing CO_2_ with H_2_ using natural proton gradients across thin, semi-conducting, inorganic barriers. Initial work reported here characterises the Fe(Ni)S precipitates and establishes sampling and detection methodologies for small organics including formate and formaldehyde.
(ii) The formation of key biochemical intermediates such as amino acids, fatty acids and sugars from methyl sulfide, CO, NH_3_ and formaldehyde. Initial work reported here characterises the synthesis of sugars including ribose from HCHO via the formose reaction, which generates sugars that could be used for RNA synthesis under alkaline hydrothermal conditions.
(iii) The concentration of organic molecules within a microporous matrix, via thermophoresis under open, continuous flow conditions. Initial work reported here demonstrates substantial temperature gradients of ~50 °C across a microporous ceramic foam (diameter 9 cm), which enable the concentration of fluorescein via thermophoresis by ~5,000-fold.

These studies are preliminary, but show the scope for more sophisticated future experiments in the reactor, and reinforce the potential of alkaline hydrothermal vents as promising far-from-equilibrium electrochemical reactors for the origin of life.

## The Reactor

### Design and Fabrication

**Fig. 2:**
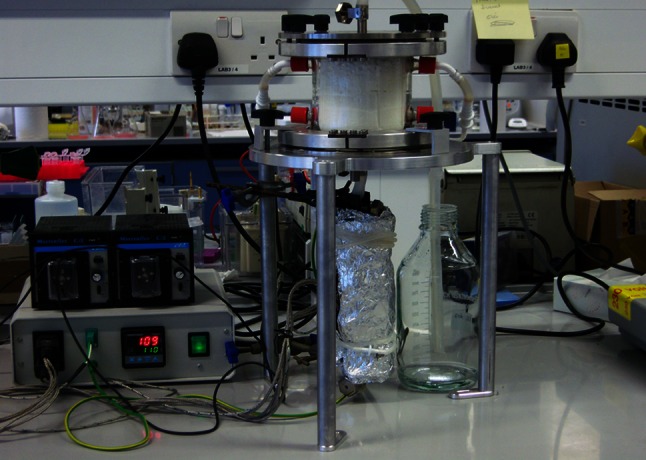
Photograph of the bench-top reactor containing ceramic foam within the reactor vessel. The reaction chamber is open-flow allowing for heated alkaline fluids and cool acidic fluids to be pumped into the main chamber with an outflow from the top into a collection vessel. There are several ports on the side of the reactor, which allow for addition of fluids or sampling while the reactor is in operation

The simple bench-top reactor simulates a continuous, open-flow, alkaline hydrothermal vent (Fig. 2). The main vessel is borosilicate glass with an internal diameter 100 mm, height 100 mm and wall thickness 5 mm. Eight side ports provide for infusion of fluids or sampling. A grade-5 titanium plate is held tightly against each end of the reaction vessel, and each plate is fitted with an inlet/outlet for the reaction vessel. A drilled titanium disc flow distributor is fitted to the inlet to distribute inflow within the reactor vessel. The external titanium inlet tube is fitted with two heating elements, and thermocouples attached to this inlet tube allow temperature control of the ‘hydrothermal’ fluids. Fluids feed into the reactor through Viton™ tubing using variable speed peristaltic pumps. Flow rates are generally set between 10 and 120 mL/h, depending on requirements. The reactor is housed in an anaerobic hood under a controlled atmosphere of 98 % N_2_/2 % H_2_ to ensure anoxia. All solutions were prepared within the anaerobic hood using HPLC-grade water that had been deoxygenated for 24 h before use.

### Precipitation of Thin, Inorganic Barriers

**Fig. 3:**
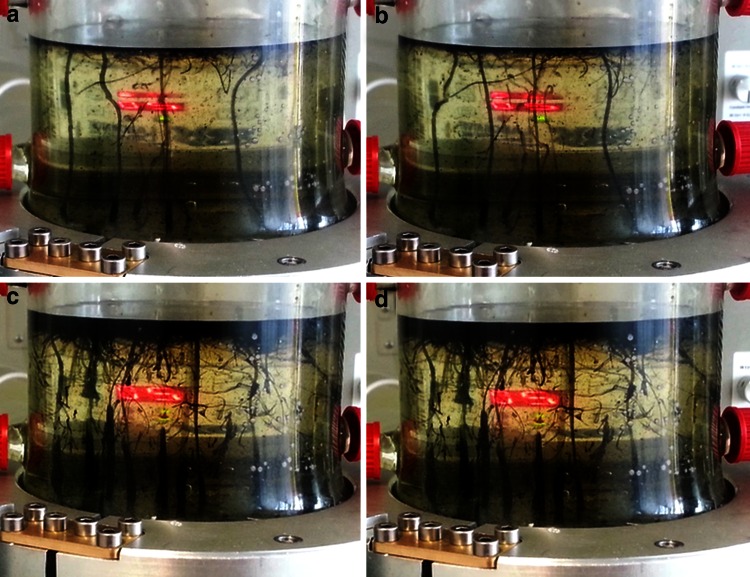
A series of photographs taken of the precipitates formed inside the reactor vessel over 4 h. **a** Initial formation of the precipitate structures. **b** After 20 min, the structures continue to form with the only disruption as they hit the surface of the fluid in the reactor. **c** After 1 h, precipitates of good structure are still forming. **d** After 4 h the precipitates become thicker around the base, probably inhibiting reduction across the barrier

To precipitate dynamic, thin-walled, semi-conducting Fe(Ni)S barriers, alkaline fluids (~pH 11, 70 °C, see Table 1 for fluid compositions) were infused into an acidic ‘ocean’ (~pH 5, 20 °C) inside the reaction vessel. Mixing of the two fluids results in the dynamic precipitation of vertical hollow tube structures composed mostly of ferrous silicates and phosphates, as well as lesser amounts of Fe(Ni)S minerals. The characteristics of these dynamic structures vary depending on the flow rates and chemical composition of the acid and alkaline fluids. To reduce CO_2_ successfully, we hypothesise that hollow thin-walled structures are needed to act as semi-conducting barriers to harness the natural proton gradient from the alkaline interior to the acidic ‘ocean’ (Fig. 3). The composition of fluids and flow rate into the reactor was varied to achieve stable, thin-walled structures. The ideal alkaline flow rate required to produce suitable structures is ~50 mL/h, which allows dynamic structures to form over several hours before thickening into more solid chimney-like structures (Fig. 3).

**Table 1:** Table giving fluid composition for both alkaline and acidic reactor fluids

Acid	Conc. (mM)	Alkali	Conc. (mM)
FeCl_2_	50	Na_2_Si_3_O_7_	10
NaHCO_3_	10	K_2_HPO_4_	10
NiCl_2_	5	Na_2_S	10

The fluids are made up in 2 L batches in millimolar concentrations as shown under strict anaerobic conditions. All water is degassed by bubbling nitrogen for 30 min prior to preparation

**Fig. 4:**
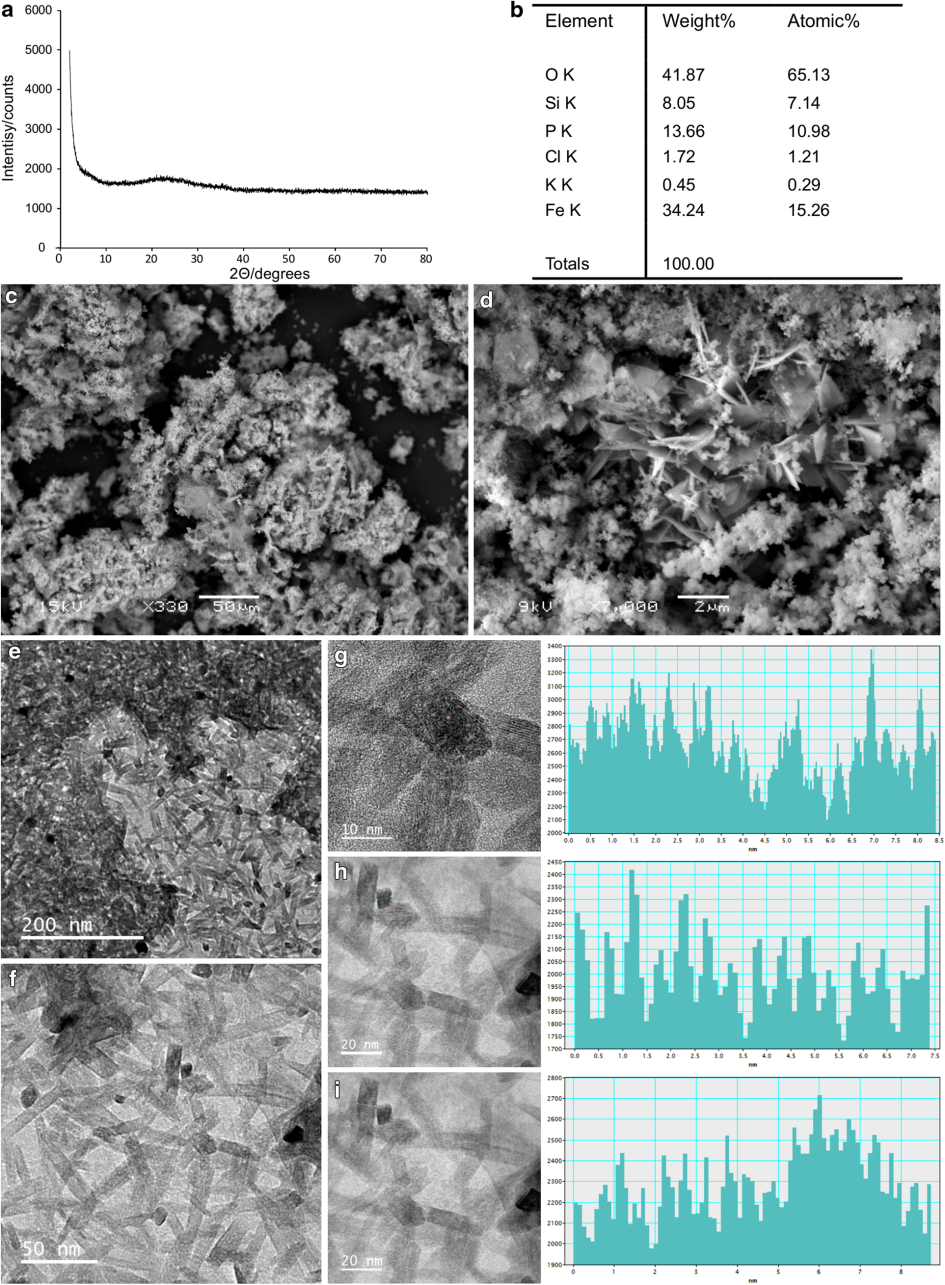
**a** Powder X-ray diffraction trace showing the precipitate is amorphous in character to X-rays: there are no peaks to indicate diffraction from crystal planes. **b** Results of elemental analysis of bulk precipitate conducted by EDX analysis. **c** SEM image of the precipitate collected from the reactor at ×330 magnification. **d** SEM image of the precipitate at ×7,000 magnification. **e** TEM image of the crystalline fractions of precipitate showing the presence of long, thin tetragonal crystals. **f** ×4 magnification of previous image showing the tetragonal crystals. **g–i** TEM lattice imaging of individual crystals showing visible atomic planes in the crystals. This planar difference was measured using a Gatan Digital Micrograph. The traces show light intensity at a specific cross-section of an individual micrograph indicating the spacing between the atomic planes. Average spacings measured were **g** 0.3 nm, **h** 0.5 nm and **i** 0.5 nm

Scanning electron microscopy (SEM) with Energy Dispersive X-ray (EDX) analysis of precipitates showed structures consisting largely of iron, phosphorus, silicon and oxygen (Fig. 4a) which are not homogeneous in composition. The precipitates are amorphous to X-rays on XRD analysis, probably because of low particle diameter. Transmission electron microscopic (TEM) inspection of some precipitates identified separate small crystalline structures (10 nm × 30 nm) from which lattice images were taken (Fig. 4g–i). The spacing of these planes was measured on the image using the profile tool in Digital Micrograph™. Of twelve crystals, spacings of 0.3, 0.5 and (occasionally) 0.7 nm were recorded. Generally, the low index planes are likely to be imaged. Jeong and coauthors (2008) reported the unit cell of mackinawite ([Fe,Ni]_1+*x*_ S, where *x* = 0 to 0.11), a tetragonal crystal structure, as *a* = *b* = 0.3 nm and *c* = 0.5 nm consistent with the presence of fine mackinawite crystals associated with some precipitates.

### Formation of Organics

**Fig. 5:**
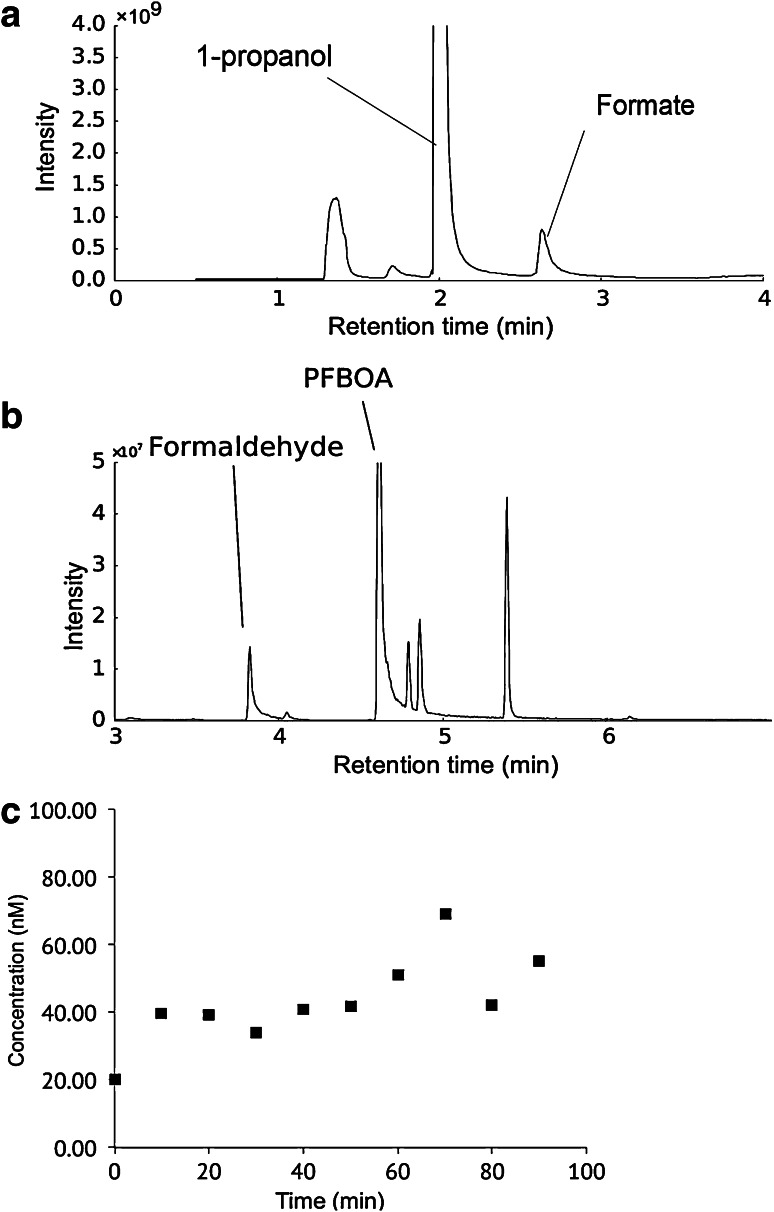
**a** GC–MS trace showing the analysis for formate. The formate peak at 2.45 min is the propyl-ester of formate. Estimated concentration is 50 µM based on extrapolation from calibration data. **b** GC–MS trace showing analysis for formaldehyde. The formaldehyde peak at 3.8 min is the PFBOA adduct. Estimated concentration is 100 nM based on extrapolation from calibration data. **c** Graph of formaldehyde concentration over time during the course of an experimental run. After an initial increase, the concentration remains relatively constant, though repeatability of sampling, total volume and dynamic reaction environment all impact on the repeatability and consistency of results

We now consider the reduction of CO_2_ under these simulated vent conditions. Fluid samples were collected close to the precipitates within the reactor every 20 min for periods of up to 4 h, and then analysed using various different methods and techniques, including high performance liquid chromatography (HPLC) and gas chromatography–mass spectrometry (GC–MS), to identify simple organics such as formate and formaldehyde. We established the presence of formate in low micromolar quantities using a head-space GC–MS analysis by derivatization of an acidified sample with 1-propanol to form the propyl-ester (Fig. 5a). We also detected formaldehyde in samples, again by head-space GC–MS analysis, in low nanomolar quantities by derivatization of the sample with *o*-(2,3,4,5,6-pentafluorobenzyl)-hydroxylamine (Fig. 5b). While these preliminary findings are encouraging, the formation of formaldehyde, especially, is variable and inconsistent between runs, and these methods are still being optimised.

### Formation of Ribose Via the Formose Reaction Under Alkaline Hydrothermal Conditions

**Fig. 6:**
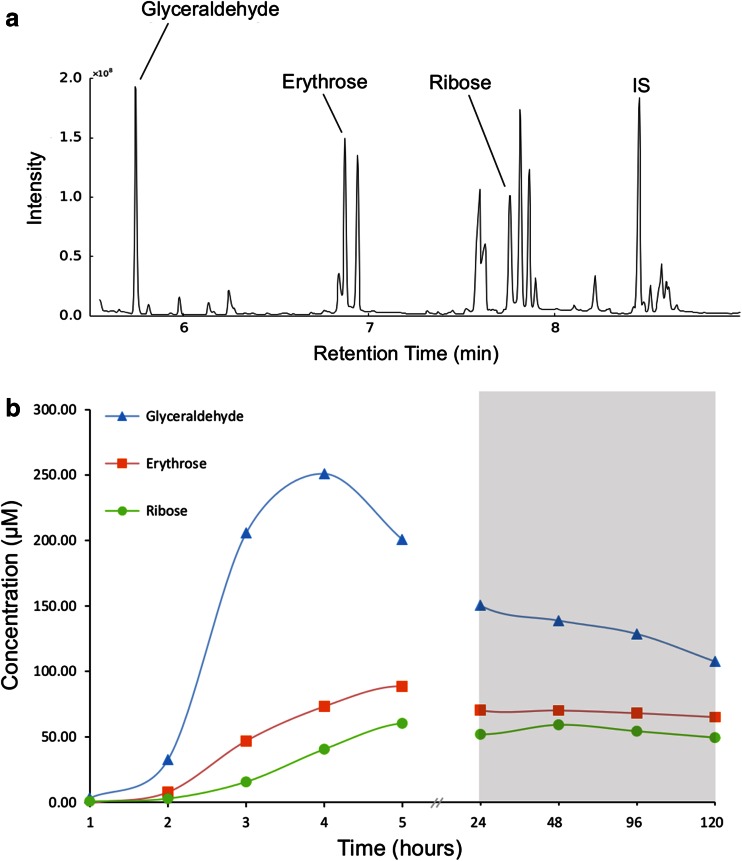
**a** GC–MS trace showing sugars, with the peaks for glyceraldehyde, erythrose and ribose labelled. The internal standard (IS) used was myo-inositol. All enantiomers of ribose (arabinose, lyxose and xylose) were identified using known standards. **b)** The concentration over time of glyceraldehyde, erythrose and ribose. The area showing 0 to 5 h is with the reaction heated to 60 °C while the shaded area shows the reaction mixture* left* at ambient temperature (~20 °C) between 5 and 120 h

The production of formaldehyde in the reactor is a proof of principle. Introducing formaldehyde at higher concentration should drive the synthesis of various sugars under simulated vent conditions via the formose reaction. Using the method of Kopetzki and Antonietti (2011), which is consistent with internal alkaline vent conditions, formaldehyde (0.5 M) was heated in alkaline fluids (initially pH 12) at 60 °C for 5 h. A variety of sugars up to C7 (septoses) were identified within the reaction fluids by GC–MS analysis (Fig. 6) after reduction with sodium borohydride and derivatization with acetic anhydride and acetic acid. Sugars identified by this process include ribose and deoxyribose, albeit at low yield (~0.06 % of formaldehyde was converted to ribose). Ribose is stable under these alkaline conditions for at least several hours, with any breakdown balanced by new synthesis. This is significant as it allows ribose to be formed under alkaline conditions, at low yields, and then potentially concentrated via thermophoresis in cooler, more neutral regions of the vent, discussed below.

### Vent Matrix

As alkaline vents are chemically varied (Lost City is mainly calcium carbonate, whereas Strýtan is constructed of saponite clay), it is difficult to establish which ions or minerals are important within the vent for promotion of prebiotic reactions, especially as early mineral structures would have been very different in the absence of oxygen and in mildly acidic oceans. We are therefore using life itself as our guide, as discussed above. A porous, ceramic foam matrix allows full control of the chemical environment. The microporous foam is constructed from alumina, which is chemically inert and has minimal catalytic properties. Depending on pH, alumina allows adsorption onto its surface and can be doped with ions or minerals relevant to early biochemistry, notably Fe(Ni)S, Mg^2+^, Ca^2+^ and Mo^4+^, as required to investigate their effects on vent chemistry.

**Fig. 7:**
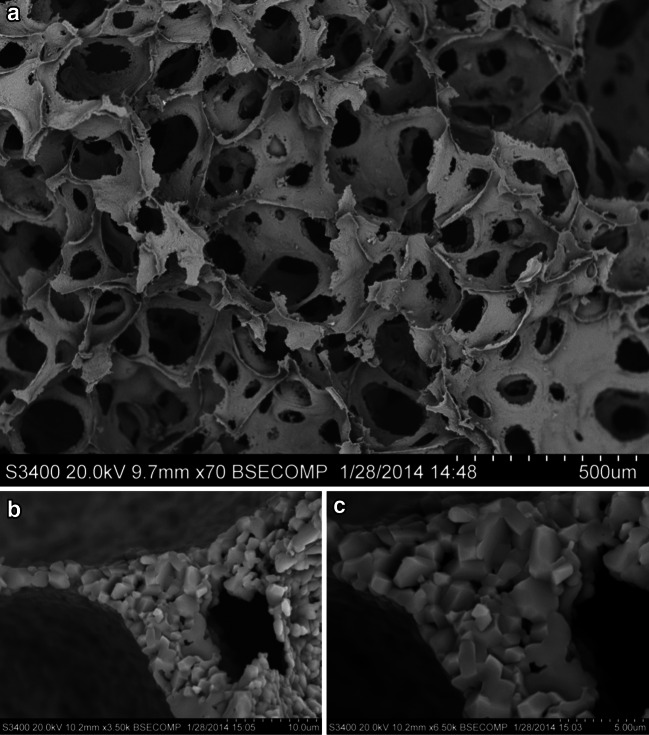
**a** SEM image of the internal structure of the ceramic foam at ×70 magnification. The foam has a microporous, highly permeable structure with interconnected cavities ~100 µm in diameter. **b** SEM image of the foam structure at ×3,500 magnification showing ~10 µm cavities within the foam structure and also sub-micron holes inside the ceramic struts of the foam. **c** SEM image of a foam strut at ×6,500 magnification showing in greater detail the sub-micron cavities in a foam strut

Foams were constructed to fit the reactor vessel, with diameter of 100 mm and height of 100 mm. The foam matrix has a ~4 % density with ~81.5 % continuous or ‘open’ porosity, meaning it has a large internal volume of interconnected pores, as in Lost City, although the morphology of our synthetic foam differs from the elongated vertical channels characteristic of the labyrinthine structure of Lost City. The ceramic foam is very hydrophilic, with water immediately and rapidly drawn in by capillary action. Analysis by SEM shows that the foam consists of a highly interconnected, porous network (100–500-µm diameters) with thin walls (1–6 µm in diameter) which themselves are highly porous with <1 µm pores in the alumina substrate (see Fig. 7 and inset).

### Temperature Gradients

**Fig. 8:**
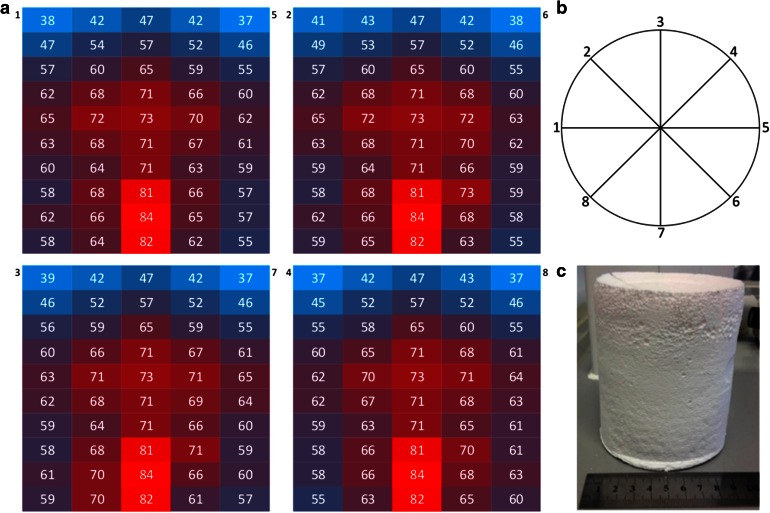
Temperature profile inside a foam exposed to vent-like conditions. The temperature graphs present axial profiles based on the division of the foam as shown (1–5, 2–6, 3–7, 4–8) in the circle diagram (*inset*). Fluid at 70 °C was flowing into the foam at a rate of 15 mL/h with a coolant fluid at 20 °C being pumped into the reactor at a rate of 120 mL/h. The reactor was left for a period of 1 h prior to temperature readings being taken. The warmer temperatures (*red*) are observed in the lower central regions with cooler temperatures (*blue*) in the upper outer regions of the foam. The photo (*inset*) shows the size of the foam before the temperature profile was taken

Ceramic foam filling the full reactor vessel was submerged in ‘ocean’ fluid at 15 °C. ‘Hydrothermal’ fluid heated to ~75 °C was pumped into the foam at ~30 mL/h via a flow distributor. Cooling fluid was pumped into the foam in the reactor vessel via 8 side ports, at ~180 mL/h to help maintain the maximum temperature gradient, while overflow was pumped out from the top. The reactor was allowed to equilibrate for 45 min before readings were taken. Temperature readings were taken from top to bottom, outside to centre along 8 axes shown in figure inset, at 10-mm depths. The temperature readings and locations are shown in Fig. 8.

The temperature profiles across the foam are as anticipated. The warmest region is at the point of inflow (centre, bottom) with cooler regions to the outside edges (where cooling fluids enter via the side ports) and the top of the foam. Fluid convection is also observed within the foam around the midpoint based on analysis of the axial temperature profiles. Temperature gradients of ≤0.5 °C mm^−1^ (i.e. up to 50 °C across the whole foam) are stable for several hours.

### Organic Concentration

We investigated whether thermophoresis can operate within the ceramic foam under open-flow conditions, using fluorescein and quinine. These compounds are UV fluorescent, so their distribution can be observed under a UV lamp, while allowing for semi-quantitative analysis of concentration. Initial testing of foam pieces soaked in standard concentrations of each indicator showed that fluorescein gives the strongest fluorescent response in the foam, but saturates at ~30 µM concentration, while quinine saturates at ~50 µM (Fig. 7). However, fluorescein is visible at concentrations >100 µM without excitation. We therefore investigated the potential thermophoretic concentration of 1 µM fluorescein.

**Fig. 9:**
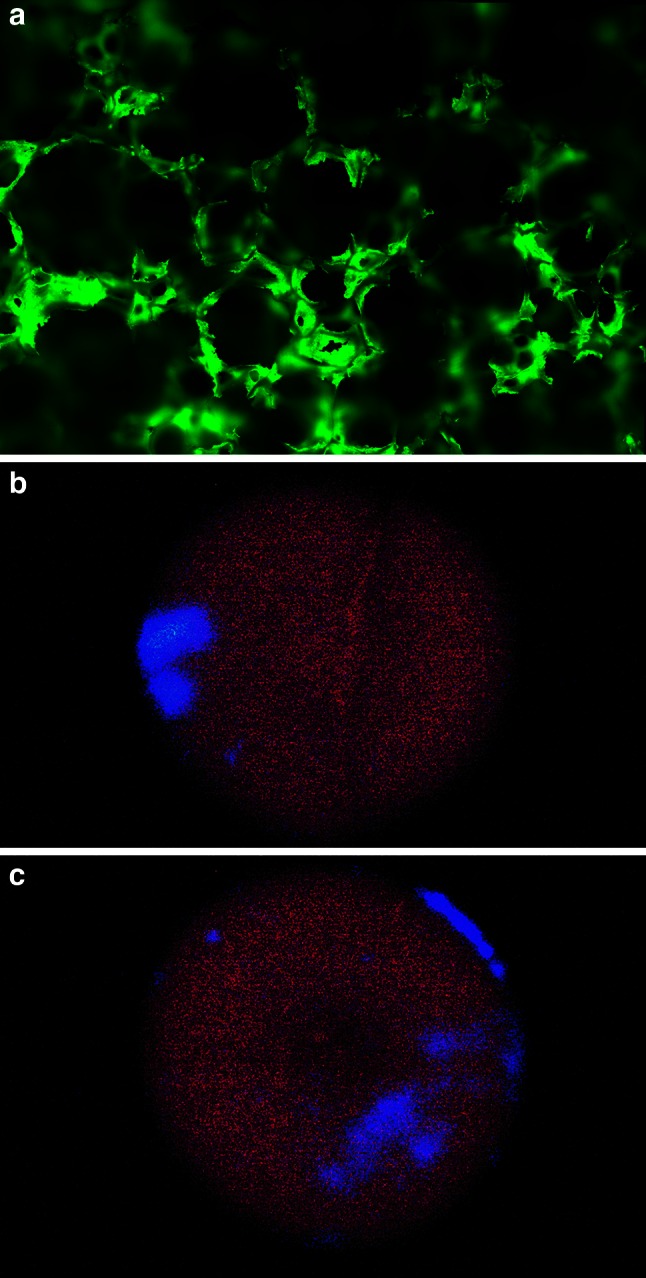
**a** Fluorescent micrograph of foam soaked in a 50 µM fluorescein solution. Fluorescein enters the struts of the foam via the sub-micron pores in the structure and remains within the foam struts, not in the cavities as originally assumed. **b** Sections of the foam exposed to UV light. The foam has been infused with 0.1 µM fluorescein solution for a period of 4 h under vent conditions. The* bright blue areas* in the photos are areas of fluorescein concentration, estimated to be between ×2,500 and ×5,000 the concentration of inflow fluids (0.1 µM)

Using standard temperature/flow conditions for thermal gradients (15 °C cooling fluids, 180 mL/h flow and 75 °C fluorescein solution, 30 mL/h flow), fluorescein (1 µM solution) was infused from the base over 7 h. Visible inspection of foams showed large regions towards the exterior (5–10-mm diameter) with intense concentration (ca. 200 and 400 µM, respectively, Fig. 9b). Thin slices of damp foam show large numbers (50–100) of 3–5-mm diameter spots in cooler regions (ca. 20–50 µM concentration). The background concentration observed is much lower than the infusion concentration. The infusion enters at 0.1 µM, not 1 µM, due to dilution by bulk ‘ocean’ fluid. It then accumulates slightly within the foam, giving a background concentration of 0.45–0.55 µM, before dramatically concentrating in the cooler regions.

These patterns suggest thermophoretic concentration. As expected, the regions of the foam with the highest concentrations are in the cooler zones (outer edges), while the warmest region close to the central base shows the weakest concentration, despite the direct entry of fluorescein into the foam at this point. Control runs without coolant or without heating failed to show any significant thermal gradients, or high concentrations of fluorescein. Fast coolant flow through the side ports precludes the external flow of fluorescein solution around the foam (which is in any case very hydrophilic, as noted). Thermophoresis of fluorescein followed by adsorption onto alumina struts in cooler regions (Fig. 9) shows that fluorescein can be concentrated up to 5,000-fold. Such high concentration may also be achievable with more prebiotically relevant molecules such as amino acids and nucleotides.

## Conclusions

We have constructed a simple bench-top reactor to investigate the possible origins of biochemistry in alkaline hydrothermal vent systems. Within the reactor vessel, thin-walled, inorganic structures precipitated, containing catalytic Fe(Ni)S microcrystals. When transected by pH gradients, these precipitates appear to be capable of reducing CO_2_ to form low yields of simple 1C organics including formate and formaldehyde. Starting with formaldehyde under mild alkaline hydrothermal conditions (60 °C, pH 11–12), a range of sugars, including ribose and deoxyribose, could be formed via the formose reaction. The concentration of these sugars is stable over several hours. While produced at low yield, organics can theoretically be concentrated by thermophoresis in a microporous matrix. We produced a simulated vent matrix from alumina foam with a highly interconnected microporous network similar to those seen in the Lost City vent system. Temperature gradients of 50 °C across this matrix can concentrate fluorescein up to ~5,000-fold under open-flow vent conditions. The studies presented here are a preliminary proof of concept, and still require optimising in many respects; they do, however, provide empirical evidence that simple organics can be generated and concentrated under mild alkaline hydrothermal conditions from H_2_ and CO_2_ using Fe(Ni)S catalysts transected by natural proton gradients in microporous matrices.
